# Changes of Major Antioxidant Compounds and Radical Scavenging Activity of Palm Oil and Rice Bran Oil during Deep-Frying

**DOI:** 10.3390/antiox3030502

**Published:** 2014-07-10

**Authors:** Azizah Abdul Hamid, Mohd Sabri Pak Dek, Chin Ping Tan, Mohd Asraf Mohd Zainudin, Evelyn Koh Wee Fang

**Affiliations:** 1 Department of Food Science, Faculty of Food Science and Technology, Universiti Putra Malaysia, UPM Serdang, Selangor 43400, Malaysia; E-Mails: mpakdek@uoguelph.ca (M.S.P.D.); asraf_zainudin@yahoo.com (M.A.M.Z); evelyn_kwf@hotmail.com (E.K.W.F.); 2 Department of Food Technology, Faculty of Food Science and Technology, Universiti Putra Malaysia, UPM Serdang, Selangor 43400, Malaysia; E-Mail: tancp@upm.edu.my

**Keywords:** vegetable oils, deep-frying, kinetic degradation, free radical scavenging, oil antioxidants

## Abstract

Changes in antioxidant properties and degradation of bioactives in palm oil (PO) and rice bran oil (RBO) during deep-frying were investigated. The alpha (α)-tocopherol, gamma (γ)-tocotrienol and γ-oryzanol contents of the deep-fried oils were monitored using high performance liquid chromatography, and antioxidant activity was determined using 2-diphenyl-1-picryl hydrazyl (DPPH) radical scavenging activity. Results revealed that the antioxidant activity of PO decreased significantly (*p* < 0.05), while that of RBO was preserved after deep-frying of fries. As expected, the concentration of α-tocopherol in PO and γ-tocotrienol in both PO and RBO decreased significantly (*p* < 0.05) with increased frying. Results also showed that γ-tocotrienol was found to be more susceptible to degradation compared to that of α-tocopherol in both PO and RBO. Interestingly, no significant degradation of α-tocopherol was observed in RBO. It is suggested that the presence of γ-oryzanol and γ-tocotrienol in RBO may have a protective effect on α-tocopherol during deep-frying.

## 1. Introduction

Vegetable oils are generally known to be healthy, due to being high in unsaturated fatty acid and other various phytochemical compounds. Palm oil (PO) is produced from the fruit bunches of the tree oil palm (*Elaeis guineensis*) and has been used for food preparations, as well as other commercial applications in many parts of the world. Yellow palm olein is well known for its high concentration of lipophilic antioxidants, in particular, tocopherols and tocotrienols, as compared to that of other vegetable oils, but lacking in carotenoids, which are removed during the refining process [1]. In addition, PO contains an equal ratio of unsaturated to saturated fatty acids, making it a versatile oil for commercial usage without involving major modification processes [2].

Rice bran oil (RBO) has been used as cooking oil in some parts of the world, particularly in Japan and India [3]. Rice bran oil has been reported to be an excellent source of antioxidants, such as tocopherols, tocotrienols and oryzanols [4]. Generally, RBO consists of up to 1.5% of oryzanol depending on the variety of the rice and is well known for its stability as a frying oil [5,6]. The role played by both tocols and oryzanol in improving lipid stability and human health is fascinating, although the use of rice bran oil is not as popular as that of other vegetables oils [7,8]. Studies have shown that tocopherols, tocotrienol, carotenoids and oryzanols exhibited potent antioxidant properties in both *in vivo* and *in vitro* systems [9,10].

Deep-frying is a convenient and rapid process to produce fried foods with intense flavor [11]. In this process, food is immersed in edible oil at a maintained temperature (150 to 200 °C) for a specific period of time [12]. However, in certain conditions, instead of the desirable intensive flavor, undesirable off-odor and toxic compounds from lipid peroxidation can also be produced during deep-frying, which is normally associated with the type of oil used. The alteration of flavor and oil quality during deep-frying can occur via the hydrolysis, oxidation and polymerization of the oil used as a result of the long exposure to high temperature [13]. Several *in vivo* studies revealed the existence of the relationship between deep-frying oil quality intake with oxidative stress level. The intake of such an altered oil quality could affect both the plasma and mitochondrial membrane [14,15].

Although oils, like PO and RBO, contain bioactive compounds that can protect lipids from oxidation, the reaction can still occur during deep-frying, which results from the degradation of the bioactive compounds, as affected by exposure to high temperature [3,13]. Therefore, it is important to evaluate the stability and degradation pattern of bioactive compounds in the oils during food preparations, in an effort to get a better picture on effective oil usage before complete depletion of bioactive compounds occur. The consumer can then gain the beneficial effects from such oils, including the lowering of serum cholesterol and blood pressure, reducing the risk of diabetic necropathy and cardio-reperfusion injury [8,16,17]. Chiou *et al.* [18] reported that during frying, foods absorb oil, the composition of which is similar to that remaining in the frying pan. Therefore, it is vital to investigate the quality of the oils during and after the frying process.

A number of studies have provided information on the quality deterioration of frying oils, but only a few studies focused on the stability of bioactive compounds during deep-frying [19,20,21]. The information regarding the antioxidant degradation kinetic and the changes of these bioactive compounds during deep-frying is relatively scarce. Hence, this study was conducted to evaluate the radical scavenging capacity and prominent bioactive compounds (α-tocopherol, γ-tocotrienol and γ-oryzanol) in PO and RBO, as well as to examine their degradation kinetic during the deep-frying of French fries.

## 2. Experimental Section

### 2.1. Materials

Yellow palm olein oil (Buruh Brand, Shah Alam, Malaysia), rice bran oil (Green Love Brand, Amorchai Co., Ltd., Bangkok, Thailand) and French fries (KG Brand, Shah Alam, Malaysia) were purchased from a local supermarket in Serdang, Selangor, Malaysia. Alpha-tocopherol, γ-tocotrienol and 2,2-diphenyl-1-picrylhydrazyl (DPPH) were purchased from Sigma-Aldrich, Steinheim, Germany. The gamma-oryzanol standard was purchased from Wako Pure Chemical Industries Ltd., Beijing, China. Hexane, isopropyl alcohol, acetonitrile, butanol, acetic acid and isooctane were purchased from Fisher Scientific, U.K. All other reagents and chemicals used were of analytical or HPLC grade.

### 2.2. Deep-Frying Model

A deep-frying model as described by Schroeder *et al.* [22] was adopted with slight modification. Frying oil with an initial volume of 3 L was heated to 180 ± 5 °C in a kitchen fryer (Philux, Model DF30AIT, Libertronic Sdn Bhd, Seri Kembangan Selangor). French fries (100 g) were then fried in the oil for 2 min, denoted as first batch deep-frying. The fried French fries were then removed, and the deep-frying was repeated with a new batch of French fries without any time lag. This procedure was repeated until the 60^th^ frying cycle. An aliquot of the oil (30 mL) was sampled after each tenth batch of French fries deep-frying to be analyzed and compared with that of fresh oil.

### 2.3. Determination of Free Radical Scavenging Capacity

The antioxidant capacity of fresh PO and RBO and after deep-frying (20^th^, 40^th^ and 60^th^ frying cycles) were determined using the 2,2-diphenyl-1-picryl hydrazyl (DPPH) radical scavenging assay as described by Lee *et al.* [23]. Briefly, 0.7 grams of oil samples were dissolved in 10 mL isooctane as a stock solution. Dissolved oil (0.25 mL) was then added to 1.75 mL DPPH solution (25 mg·L^−1^ prepared in isooctane). The mixture was then vortexed and incubated at ambient temperature for 30 min. The absorbance was measured at 515 nm using a UV-Vis spectrophotometer (EL800, Biotek, Winooski, VT, USA). The radical scavenging activity was calculated using the following formula:

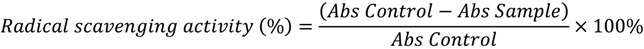

where Abs control is the absorbance of the control reaction (containing all reagents except the test compound) and Abs sample is the absorbance of the test sample. Synthetic antioxidant butylated hydroxyanisole (BHA) and α-tocopherol were used as positive controls.

### 2.4. Determination of α-Tocopherol and γ-Tocotrienol

Alpha-tocopherol and γ-tocotrienol were determined according to the American Oil Chemists’ Society (AOCS) Ce 8-89 [24] method with slight modifications. Oil was dissolved with 10 mL of hexane:isopropyl alcohol (ratio 98:2) and filtered through a 0.45 μm (Whatman) nylon membrane filter prior to injecting into a HPLC. An aliquot of sample (20 μL) was analyzed using a normal phase HPLC system equipped with chromatography software (Millennium LC-6A, Shimadzu, Kyoto, Japan), a UV detector (SPD-6A, Shidmazu, Kyoto, Japan) and pumps. The separation was performed with an ACE 5 Silica column (250 × 4.6 mm, 5 μm) operated at ambient temperature. The mobile phase consisted of hexane:isopropyl alcohol at a ratio of 98:2 (v/v) with a flow rate of 1 mL·min^−1^, and the detector was set at 295 nm. The identification of unknown tocopherol and tocotrienol isomers was based upon matching their retention time with those of pure standards, while the concentrations calculated using the peak area based on standard calibration curves.

### 2.5. Determination of γ-Oryzanol in Rice Bran Oil

The content of γ-oryzanol was determined according to the method of Xu and Godber [25]. An aliquot (0.1 g) of oil was dissolved in 10 mL hexane:isopropyl alcohol at a ratio of 98:2 (v/v). The dissolved oil sample was then filtered through a 0.45 μM (Whatman) nylon membrane filter prior to injecting into a HPLC. The filtered sample (20 μL) was analyzed using an HPLC system (Millennium LC-6A, Shimadzu, Tokyo, Japan) chromatography manager, a UV detector (SPD-6A, Shidmazu, Tokyo, Japan) and pumps. The separation was carried using a Waters reverse-phase (RP) μBondapak C18 column (250 × 4.6 mm, 5 μM; Waters Corp., Milford, MA, USA) at ambient temperature. The mobile phase consisted of acetonitrile/butanol/acetic acid/water at a ratio of 94:3:2:1 (v/v/v/v). The flow rate was kept at 1 mL·min^−1^ with the isocratic mode, and the detector was set at 330 nm. Identification of the γ-oryzanol component in the RBO was done by comparing its retention with that of pure standard, and quantification was done based on the standard calibration curve.

### 2.6. Determination of Degradation Kinetics Using the Order of Reaction Equation

According to Taoukis *et al*. [26], the degradation kinetics of many compounds in foods at constant temperature follows the first-order kinetics model, which can be expressed as follows:

−dC/dt = kC
(1)

Integration of Equation 1 gives:

lnC = lnCo − kt
(2)

lnC/Co = −kt
(3)

where C is the concentration of the compound, Co is the initial concentration of the compound, t is time and k is the reaction rate constant. By plotting the logarithm of the concentration of α-tocopherol and γ-tocotrienol, respectively, over the initial concentration against a number of frying cycles during the degradation process, the degradation rate constant for each compound was calculated from the slope of the simple linear regression line.

### 2.7. Statistical Analysis

All frying experiments and analyses were conducted in triplicate. Data was analyzed statistically through one-way ANOVA by Duncan’s multiple range tests using the commercially available software, the SPSS 16 software program (SPSS Inc., Chicago, IL, USA). A *p*-value of less than 0.05 (*p* < 0.05) was considered to denote statistical significance.

## 3. Results and Discussion

### 3.1. Antioxidant Capacity of the Oils

**Figure 1:**
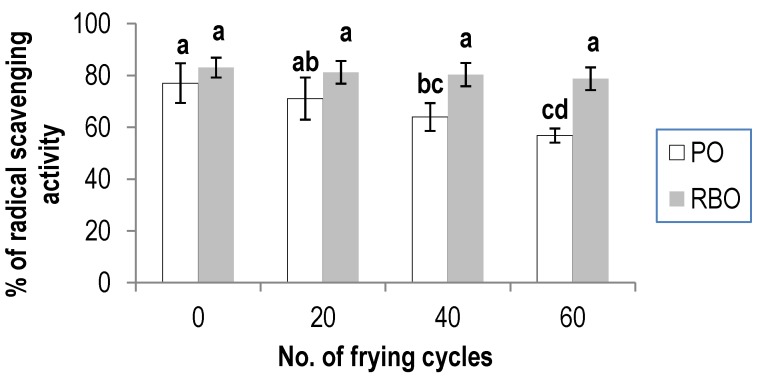
The percentage of radical-scavenging activity in palm oil and rice bran oil over the 60th batch of deep-frying at a concentration of 70 mg/mL. The values given are means ± standard deviation of a triplicate analysis. The values marked with the same letters are not significantly different at *p* < 0.05 analyzed using the Duncan multiple range test. PO, palm oil; RBO, rice bran oil.

In this study, free radical scavenging activity utilizing the DPPH radical was done in determining the antioxidant capacity of the oils, due to its simplicity and wide use in antioxidant research. The free radical scavenging capacity of PO and RBO after the 20th, 40th and 60th frying cycle is shown in Figure 1. Results showed that there is no significant difference in the antioxidant capacity between the fresh and the 20th frying cycle PO. However, a significant (*p* < 0.05) decrease in the activity was observed for the oil from the 40th and 60th frying cycle. On the other hand, there was no significant difference in the antioxidant activity exhibited by all the batches of RBO. This indicates that RBO is more stable compared to that of PO upon deep-frying. It was also noted that RBO derived from the 40th and 60th frying cycle exhibited significantly (*p* < 0.05) higher radical scavenging activity than that of PO.

The radical scavenging assay is one of the common methods that can be used to determine the antioxidant capacity of vegetable oils [27]. The results of the study showed that both PO and RBO exhibited good antioxidant capacity. However, the antioxidant capacity of PO decreased with deep-frying. This is probably attributed to the degradation of bioactive compounds as a result of the exposure to high temperature used during deep-frying. Results presented here are in line with that of a previous study conducted by Gomez-Alonso *et al.* [28], where the reduction of antioxidant activity of olive oil correlated well with the number of deep-frying cycles. However, similar the destruction of some of these bioactive compounds may not be occurring in RBO, as the antioxidant activity in the oil was preserved throughout the frying process.

### 3.2. Degradation of α-Tocopherol and γ-Tocotrienol during Deep-Frying

**Figure 2:**
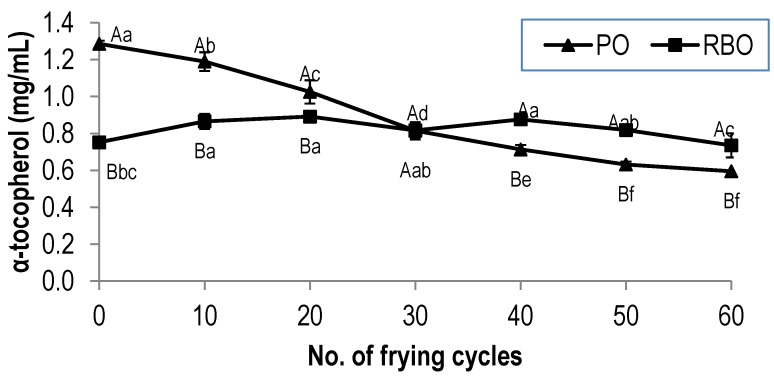
The degradation of α-tocopherol in palm oil and rice bran oil during deep-frying. The values given are means ± standard deviation of triplicate analysis. The values marked with the same letters are not significantly different at *p* <0.05 analyzed using the Duncan multiple range test. A,B, the values with different capital letters indicate a significant difference between the type of oil at *p* < 0.05. a,b, the values with different small letters indicate a significant difference between frying cycle numbers at *p* < 0.05.

The degradation of α-tocopherol in PO and RBO during deep-frying is shown in Figure 2. As expected, results showed that the concentration of α-tocopherol in PO decreased significantly with deep-frying. Similar to that of its antioxidant activity, α-tocopherol content decreased from 1.29 mg·g^−1^ to 0.59 mg·g^−1^ after the 60th frying cycle, depicting a loss of 54%. Surprisingly, α-tocopherol in RBO was found to be more stable and remained almost unaffected upon deep-frying up to the 60th frying cycle. The contents of α-tocopherol in fresh RBO and the 60th frying cycle were 0.75 mg·g^−1^ and 0.73 mg·g^−1^, respectively, a loss of only 2%. This is reflected in the antioxidant activity exhibited by the oil. The degradation of α-tocopherol in PO is in agreement with that of previous literature [14,15]. Battino *et al.* [14] found that α-tocopherol in olive oil was degraded up to 28% from the initial value after frying for 60 min.

**Figure 3:**
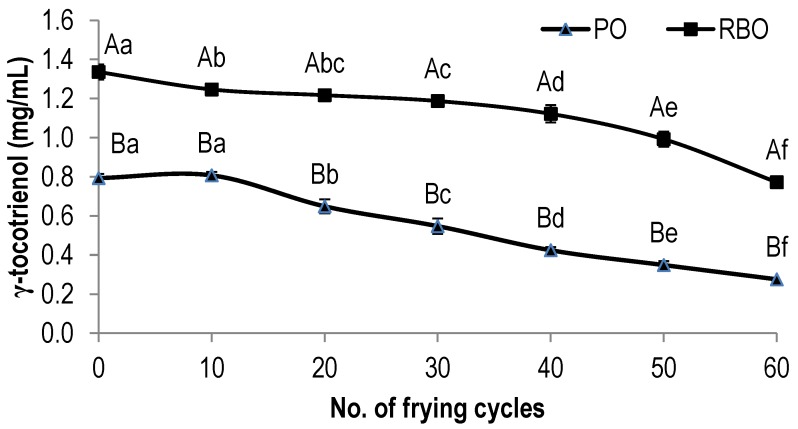
The degradation of γ-tocotrienol in palm oil and rice bran oil during deep-frying. The values given are means ± standard deviation of a triplicate analysis. The values marked with the same letters are not significantly different at *p* < 0.05 analyzed using the Duncan multiple range test. A,B, the values with different capital letters indicate a significant difference between the type of oil at *p* < 0.05. a,b, the values with different small letters indicate a significant difference between frying cycle numbers at *p* < 0.05.

Figure 3 showed the degradation of γ-tocotrienol in deep-fried PO and RBO. There was a similar trend on the loss of γ-tocotrienol in both oils during the deep-frying of French fries, where it decreased significantly with frying. However, interestingly, γ-tocotrienol’s content in RBO was always higher than that of PO in all batches of oils. The fresh PO and the 60th frying cycle PO consisted of 0.79 mg·g^−1^ and 0.28 mg·g^−1^ tocotrienol, respectively depicting a loss of 65%. Whereas, the content of γ-tocotrienol in fresh RBO and the 60th frying cycles of RBO were 1.34 mg·g^−1^ and 0.77 mg·g^−1^, respectively, showing a lower loss of 42%.

During the deep-frying of fries, the decrease in the concentration of tocopherols and tocotrienols in PO might have occurred due to their protective role against oxidation and degradation upon exposure to high temperature. A similar observation has been reported by Hoffmann [29], where at a high temperature, tocopherols and tocotrienols tend to protect the oil by oxidizing themselves to quinones and dimers. The present result also indicated that α-tocopherol and γ-tocotrienol in PO degraded at a faster rate than that of RBO. Generally, PO consisted of an equal amount of saturated fatty acid to unsaturated fatty acid, whereas RBO contained a higher amount of unsaturated fatty acid, usually up to 74%. A higher proportion of unsaturated fatty acids in RBO, relative to PO, could be correlated to the greater number of double bonds in the former oil [21,22]. Therefore, it can be suggested that during the deep-frying of RBO, these unsaturation might have competed with tocopherols and tocotrienols as substrates for oxidation, resulting in a slower degradation of these antioxidants. In addition, a higher degree of unsaturation, as a basis of more competitiveness of the fatty acids towards oxidation, may explain the high degradation rate of α-tocopherol and γ-tocotrienol in PO as compared with that of RBO [30]. This is in agreement with that reported by Hoffmann [29], who showed that during the propagation phase of oxidation, the fatty acid peroxy free radicals may react preferentially with the phenolic hydrogen molecule of tocopherol. Seppanen *et al.* [31] reported that the antioxidant activities of tocopherols and tocotrienols in oils are mainly due to their abilities to donate phenolic hydrogen to reactive lipid free radicals, thus retarding the normal steps of autocatalytic lipid peroxidation. Tocopherols can effectively scavenge peroxy radicals and yield relatively stable products, which interrupt the propagation stage of the oxidative chain reaction, thereby preventing the destruction of lipid molecules [32,33].

It is interesting to note that the concentration of α-tocopherol in RBO was maintained during the deep-frying of the fries exhibiting only a low percent loss (Figure 2). In contrast, the content of γ-tocotrienol in RBO decreased considerably with increased frying. In RBO, γ-tocotrienol is expected to possess higher antioxidant activity than that of α-tocopherol. This was also applied to PO, which exerts the same trend [22]. Therefore, it can be suggested that γ-tocotrienol was firstly oxidized in order to protect other weak antioxidants. This is in agreement with that reported by Rossi *et al.* [30], where among vitamin E homologs, γ-tocotrienol was found to be the least stable and easily oxidized during deep-frying, which might be attributed mainly to its protective role and self-degradation in preserving other homologues, such as tocopherols. Apart from that, the initial content of γ-tocotrienol in fresh RBO was significantly higher than that in PO, although the concentration of both decreased with an increase in the number of frying cycles. Therefore, with a higher concentration, the protective effect of γ-tocotrienol in preserving α-tocopherol was evident.

### 3.3. Degradation of γ-Oryzanol in Rice Bran Oil during Deep-Frying

**Figure 4:**
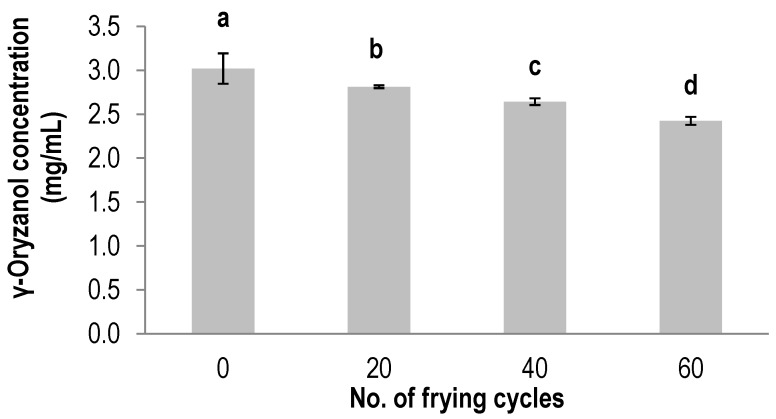
The degradation of γ-oryzanol in RBO during deep-frying. The values given are means ± standard deviation of a triplicate analysis. The values marked with the same letters are not significantly different at *p* < 0.05 analyzed using the Duncan multiple range test.

Figure 4 showed the degradation of γ-oryzanol in RBO over the 60th frying cycles. The concentration of γ-oryzanol in RBO was seen to decrease significantly (*p* < 0.05) in accordance to the number of frying batches. Fresh oil, containing 3.0 mg·g ^−1^ γ-oryzanol, was reduced to 2.8 mg·g^−1^ at the 20th cycles, 2.6 mg·g^−1^ after 40 cycles and 2.4 mg·g^−1^ after 60 batches. The remaining γ-oryzanol content in RBO after the 60th frying cycles was estimated to be 80% of the original amount in the fresh oil.

RBO naturally contains α-tocopherol, γ-tocotrienol and γ-oryzanol, making it a suitable model to study the synergistic interaction between these bioactive compounds in protecting lipids from oxidation. The ability of a compound to inhibit the oxidation process is via several mechanisms. Gamma-oryzanol is one of the most potent antioxidant compounds reported. It is interesting to note that besides acting as an antioxidant to prevent oil from oxidation, γ-oryzanol is also associated with decreasing cholesterol in plasma, platelet aggregation and cholesterol absorption [34,35,36]. In addition, γ-oryzanol has also been used to treat nerve imbalance and disorders of menopause in women [37].

The results in this study indicated that γ-oryzanol in RBO was degraded with the number of frying cycles, and its degradation is parallel to that of γ-tocotrienol. However, the α-tocopherol content in RBO did not decrease significantly (*p* < 0.05) until the 60th frying cycles (Figure 2). Thus, it can be assumed that, in RBO, γ-oryzanol and γ-tocotrienol synergistically protect α-tocopherol from degradation during deep-frying. The presence of γ-oryzanol in RBO may confer a protective effect for α-tocopherol, thus slowing down its degradation compared to that in PO. This observation was in agreement with that of Kochhar [38], who reported that γ-oryzanol has substantial synergistic effects with tocopherols. Similarly, the higher amount of γ-oryzanol, due to the addition of RBO to soybean oil, was related to the synergistic antioxidant effects in preserving tocopherol degradation during the frying of dough [39].

RBO usually contains oryzanol up to 1.5% and is well known for its good stability as a frying oil [6]. Even though RBO contained a higher percent of γ-oryzanol in comparison to that of α-tocopherol and γ-tocotrienol, the main reaction that caused the degradation of γ-oryzanol during deep-frying is due to the oxidation process [40]. In the present study, the antioxidant capacity of PO as measured by DPPH decreased significantly (*p* < 0.05) after deep-frying, similar to its α-tocopherol and γ-tocotrienol degradation. In contrast, the antioxidant capacity of RBO did not decrease significantly over 60 frying cycles. The slow degradation of γ-tocotrienol and the retention of α-tocopherol in RBO was probably attributed to the preservation of its antioxidant activity. This is in agreement with the findings of Nanua *et al.* [41], who reported that γ-oryzanol can improve the stability of RBO and acts as a natural antioxidant to improve the stability of several fried foods. Hence, it is suggested that blending PO with RBO might be helpful to preserve the tocol content during deep-frying.

### 3.4. Degradation Kinetic of α-Tocopherol and γ-Tocotrienol during Deep-Frying

**Figure 5:**
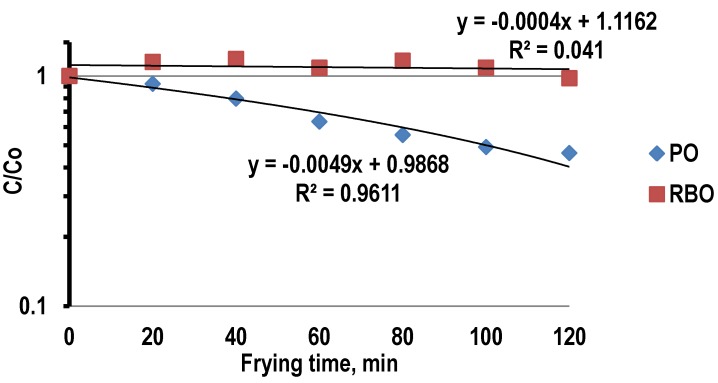
First order kinetic plots of the degradation of α-tocopherol in palm oil and rice bran oil during deep-frying.

**Figure 6:**
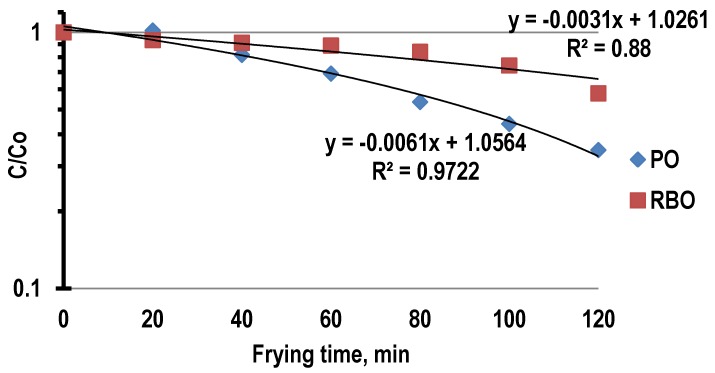
First order kinetic plots of the degradation of γ-tocotrienol in palm oil and rice bran oil during deep-frying.

The first order kinetic plots of the degradation of α-tocopherol and γ-tocotrienol in PO and RBO during deep-frying of French fries are presented in Figure 5 and Figure 6, respectively. The kinetic constant of degradation (*k*) of α-tocopherol and γ-tocotrienol and the determination of the coefficient (*R*^2^) for PO and RBO during deep-frying is shown in Table 1.

The results showed that the first-order kinetics model can be applied to approximately describe the degradation reaction of α-tocopherol in PO, but not in RBO. It is reported that the degradation kinetics of many compounds in foods at constant temperature will follow the first-order kinetics model [26]. This is in good agreement with the findings of Sabliov *et al.* [42], where heat during frying caused the degradation of free α-tocopherol followed first order kinetics and holding oils at 180 ± 5 °C showed the greatest degradation rate.

**Table 1:** Kinetic constant (*k*) of degradation of α-tocopherol and γ-tocotrienol and the coefficient of determination (*r*^2^) of palm oil and rice bran oil during deep-frying.

Variable	*k* (min^−1^)	Coefficient of Determination, (*r*^2^)
α-tocopherol in palm oil	0.004	0.961
α-tocopherol in rice bran oil	0.000	0.041
γ-tocotrienol in palm oil	0.006	0.972
γ-tocotrienol in rice bran oil	0.003	0.880

In the present study, the reaction rate constant for the degradation of both α-tocopherol and γ-tocotrienol upon deep-frying can be obtained using the order of reaction equation. The reaction rate constants of the degradation of α-tocopherol in PO and RBO were found to be 0.009 min^−1^ and 0 min^−1^, respectively and their correlation coefficients (*r*^2^) were found to be 0.961 and 0.041, respectively.

Similarly, γ-tocotrienol in PO and RBO obeyed the first-order kinetics with the reaction rate constants of degradation being 0.0061 min^−1^ and 0.0031 min^−1^, respectively, and their correlation coefficients were found to be 0.972 and 0.88, respectively. The results are in line with that of previous studies that showed that tocopherols and tocotrienols decrease as a function of time and temperature, following first-order kinetics [43]. Results from the present study showed that γ-tocotrienol in PO (*k* = 0.0061 min^−1^) degraded faster than that of α-tocopherol in PO (*k* = 0.0049 min^−1^). In addition, it was reported that the antioxidant property of γ-tocotrienol is better than that of α-tocopherol [22]. Similarly, the antioxidant capacity of γ-tocotrienol under heating conditions in stripped oils is better than that of α-tocopherol [44]. Therefore, it could be suggested that the faster degradation of γ-tocotrienol could be due to its greater antioxidant activity.

## 4. Conclusions

Based on the present study, it is concluded that PO and RBO are good sources of natural antioxidants that include tocols and oryzanols. These compounds exhibited degradation at a faster rate in PO compared to that of RBO, with the degradation kinetics obeying the first-order kinetics. It was also noted that α-tocopherol does not degrade to a significant extent in RBO by virtue of the presence of γ-oryzanol, indicating the synergy between the bioactive compounds in maintaining the nutritional value of the oil.
